# Hyperoxia-induced bronchopulmonary dysplasia: better models for better therapies

**DOI:** 10.1242/dmm.047753

**Published:** 2021-02-23

**Authors:** Kiersten Giusto, Heather Wanczyk, Todd Jensen, Christine Finck

**Affiliations:** 1 Department of Pediatrics, University of Connecticut Health Center, Farmington, 06106 CT, USA; 2 Department of Surgery, Connecticut Children's Medical Center, Hartford, CT, USA

**Keywords:** Bronchopulmonary dysplasia, Stem cell therapy, Hyperoxia

## Abstract

Bronchopulmonary dysplasia (BPD) is a chronic lung disease caused by exposure to high levels of oxygen (hyperoxia) and is the most common complication that affects preterm newborns. At present, there is no cure for BPD. Infants can recover from BPD; however, they will suffer from significant morbidity into adulthood in the form of neurodevelopmental impairment, asthma and emphysematous changes of the lung. The development of hyperoxia-induced lung injury models in small and large animals to test potential treatments for BPD has shown some success, yet a lack of standardization in approaches and methods makes clinical translation difficult. *In vitro* models have also been developed to investigate the molecular pathways altered during BPD and to address the pitfalls associated with animal models. Preclinical studies have investigated the efficacy of stem cell-based therapies to improve lung morphology after damage. However, variability regarding the type of animal model and duration of hyperoxia to elicit damage exists in the literature. These models should be further developed and standardized, to cover the degree and duration of hyperoxia, type of animal model, and lung injury endpoint, to improve their translational relevance. The purpose of this Review is to highlight concerns associated with current animal models of hyperoxia-induced BPD and to show the potential of *in vitro* models to complement *in vivo* studies in the significant improvement to our understanding of BPD pathogenesis and treatment. The status of current stem cell therapies for treatment of BPD is also discussed. We offer suggestions to optimize models and therapeutic modalities for treatment of hyperoxia-induced lung damage in order to advance the standardization of procedures for clinical translation.

## Abstract (precis)

**Summary:** Current animal models of bronchopulmonary dysplasia suffer from a lack of standardization, which hampers the translational relevancy of findings. Here the current state of available models is reviewed, offering recommendations for improvement.

## Introduction

Bronchopulmonary dysplasia (BPD) is a chronic lung disease that is multifactorial in nature. It is the most common complication of preterm birth, occurring in 43% of infants born at or before 28 weeks gestation (Lignelli et al., 2019; Willis et al., 2018). BPD is characterized by arrested lung growth, alveolar simplification, impaired blood vessel development and abnormal pulmonary function (Baraldi and Filippone, 2007). It is associated with significant morbidity and mortality in premature infants, and survivors often exhibit persistent long-term effects into adulthood (Silva et al., 2015). Disease severity has a significant sex bias, whereby preterm males have a higher risk of developing more-severe symptoms compared to females of the same age (Collaco et al., 2017).

BPD incidence has increased owing to advances in neonatal care that improved the survival of lower gestational age infants. The condition has first been described by Northway et al. in 1967 (Northway et al., 1967). The ‘classic’ BPD, determined by intense inflammation, fibrosis and scarring of the lung, has now evolved to the ‘new’ BPD, determined by reduced alveolar development together with airway injury, inflammation and mild fibrosis (McEvoy and Aschner, 2015; Mosca et al., 2011). The severity of lung injury is determined by the duration of excess O_2_ exposure and the degree of positive pressure during mechanical ventilation (Gibbs et al., 2020; Mann and Bär, 2011). Lung damage can range from early developmental arrest in patients diagnosed with new BPD to structural damage of the immature lung in neonates diagnosed with classic BPD (Mosca et al., 2011).

**Fig. 1.**
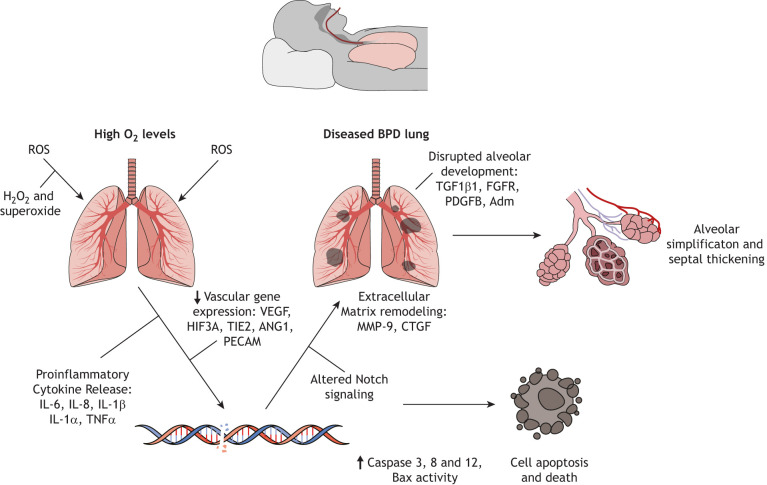
**Molecular and cellular alterations associated with BDP.** Hyperoxic conditions associated with the development of BPD lead to a release of reactive oxygen species (ROS), such as hydrogen peroxide (H_2_O_2_) and superoxide (O_2_^−^), which trigger pro-inflammatory cytokine expression. This contributes to molecular and cellular damage within the lung, including damage to DNA. Additionally, pathways associated with vasculogenesis and alveolar development, as well as Notch signaling, are dysregulated, resulting in remodeling of the extracellular matrix, apoptosis and aberrant lung development. Morphological characteristics of disease include alveolar simplification and septal thickening.

Exposure to high concentrations of O_2_ is one of the most important factors in the development of BPD. It leads to the release of reactive oxygen species (ROS), which cause significant cellular and molecular damage to the lung (Wang and Dong, 2018) (see Fig. 1). Preterm infants are at high risk of developing BPD owing to their need for O_2_ supplementation, since their immature lungs do not provide sufficient gas exchange (Chao et al., 2018). BPD pathogenesis involves a disruption of alveolar epithelial-mesenchymal signaling, causing alveolar lipofibroblasts to transdifferentiate into myofibroblasts. The latter lack the ability to maintain epithelial cell growth and differentiation, which results in reduced alveolarization (Hwang and Rehan, 2018). Premature infants are usually diagnosed with BPD 14–30 days after birth. Physical symptoms of disease include wheezing, rapid and labored breathing, repeated lung infections and difficulty in feeding (Principi et al., 2018). Currently, therapies, such as antenatal steroids, surfactant replacement and modified ventilation approaches, only treat the short-term effects of BPD. Preventative strategies include the use of lower inspired O_2_ concentrations, non-invasive ventilation strategies such as continuous positive airflow pressure and improved resuscitation at birth. However, the incidence of BPD in premature infants born before 29 weeks gestational age remains at ∼40% (Mandell et al., 2019). Currently, no treatments reverse or prevent the alveolar damage following hyperoxia.

The aim of this Review is to call attention to issues of modelling BPD induced by high levels of oxygen (hyperoxia) in animals, with a focus on outcome measurements. We discuss variations in the modelling of hyperoxia-induced BPD together with lung injury measurements, and highlight the use of *in vitro* models to evaluate the cellular and molecular pathways to show how these systems may complement current animal models. The status of potential therapies for treatment of BPD is also reviewed, followed by suggestions for the standardization of current methodologies to improve the therapeutic management of BPD in neonates.

## Lung development and BPD

**Fig. 2.**
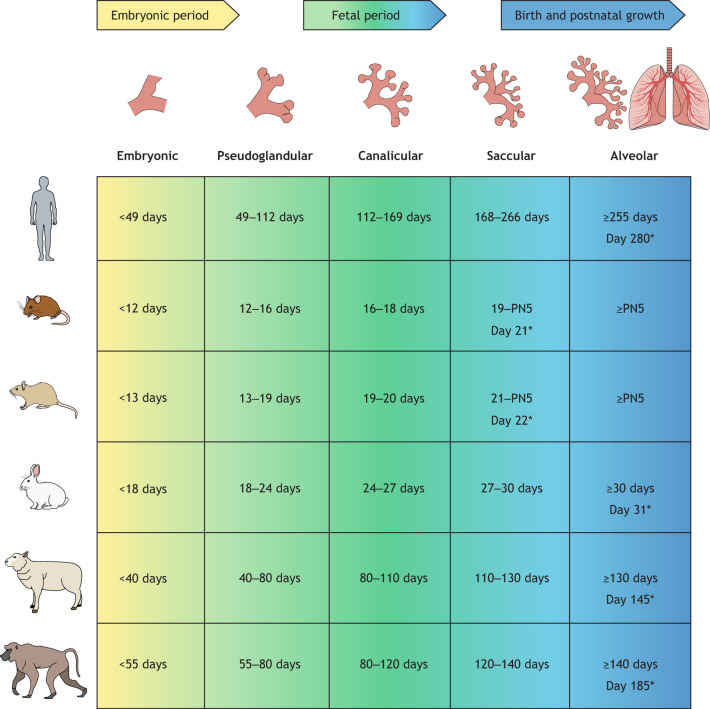
**Stages of lung development in humans and in animal species often used as disease models.** Although lung development is conserved across mammals, the individual developmental stages occur at different gestational age, which has implications for modeling pulmonary disorders, such as BPD. Stages of lung development are based on morphological criteria and do not have strict time lines; data were summarized from previous publications (Berger and Bhandari, 2014; Lau and Kavlock, 1994; Pinkerton and Joad, 2000; Schittny, 2017). PN, postnatal day; *, day when full-term gestation is reached.

In order to develop a disease model, it is important to understand both embryology and how the various animal models may relate to a human disease. There are five distinct stages to lung development that are conserved across species, and are categorized as embryonic, pseudoglandular, canalicular, saccular and alveolar (Schittny, 2017). Each stage entails defined anatomical and physical changes necessary for lung maturation, and the duration of each stage differs between species (Berger and Bhandari, 2014). Fig. 2 provides a timeline for the various stages of lung development in different species compared to those in humans. Premature infants susceptible to BPD are born during the canalicular-saccular stage of lung development, whereby branching morphogenesis and alveolar differentiation need yet to be completed (Thébaud et al., 2019).

### Morphological changes

In response to high levels of O_2_ and to mechanical ventilation, the lung elicits a pro-inflammatory response that stimulates the release of free radicals. This disrupts alveolar architecture and results in simplified structures, fewer alveoli and a reduced surface area for gas exchange (Shahzad et al., 2016). Additionally, mild airway smooth muscle thickening develops together with alveolar septal fibrosis (Kalikkot Thekkeveedu et al., 2017). This significantly impairs lung function and, in severe cases, alters pulmonary vascular development. In ∼25% of preterm infants diagnosed with BPD, pulmonary hypertension is a common finding (Shahzad et al., 2016). Lung biopsies from adolescents with a history of prematurity and BPD revealed peribronchial fibrosis and thickening of the submucosal layers, consistent with chronic obstructive lung disease and chronic abnormalities of the angiogenic process. These findings indicate that the morphological effects of BPD can persist into adulthood (Galderisi et al., 2019).

### Molecular changes

A number of studies have indicated that expression levels of cytokines, chemokines, growth factors, apoptotic factors and proteases change profoundly during hyperoxia in the neonate and in relevant animal models of BPD (summarized in Table 1 and references therein). These changes are reflected in morphological and cellular perturbations seen in the lungs of patients with severe symptoms of BPD. Hyperoxia can lead to the initiation of an inflammatory cascade within immature lung tissue, thereby interfering with the normal course of septal and alveolar development, and often precedes clinical symptoms (Lignelli et al., 2019). Increased mRNA expression of pro-inflammatory cytokines has been identified in airway secretions and bronchoalveolar cells from human neonates with developing BPD (Köksal et al., 2012). One of the most important cytokines implicated in the pathogenesis of BPD is transforming growth factor β1 (TGFβ1), a multifunctional cytokine that activates fibroblast differentiation into myofibroblasts, regulates wound healing, and can control cell growth, proliferation, differentiation and apoptosis (Martin et al., 2016). Expression of TGFβ1 inhibits alveolar development and causes pulmonary fibrosis in lungs with advanced alveolarization. In human neonates with BPD, the TGFβ1 concentration in bronchoalveolar lavage fluid is increased and used as a predictor of BPD severity (Kwong et al., 2006). Moreover, transfer of the active *Tgfb1* gene to the lungs of neonatal rats shows areas of interstitial fibrosis and enlarged alveolar spaces (Gauldie et al., 2003). This suggests that overexpression of *Tgfb1* during the crucial period of postnatal rat lung alveolarization gives rise to pathological, biochemical, and morphological changes consistent with BPD. Expression levels of *Tgfb1* mRNA increase after hyperoxia in mice and rats, and significantly decrease after treatment with mesenchymal stem cells (MSCs) (Luan et al., 2015; Chang et al., 2011).

**Table 1.**
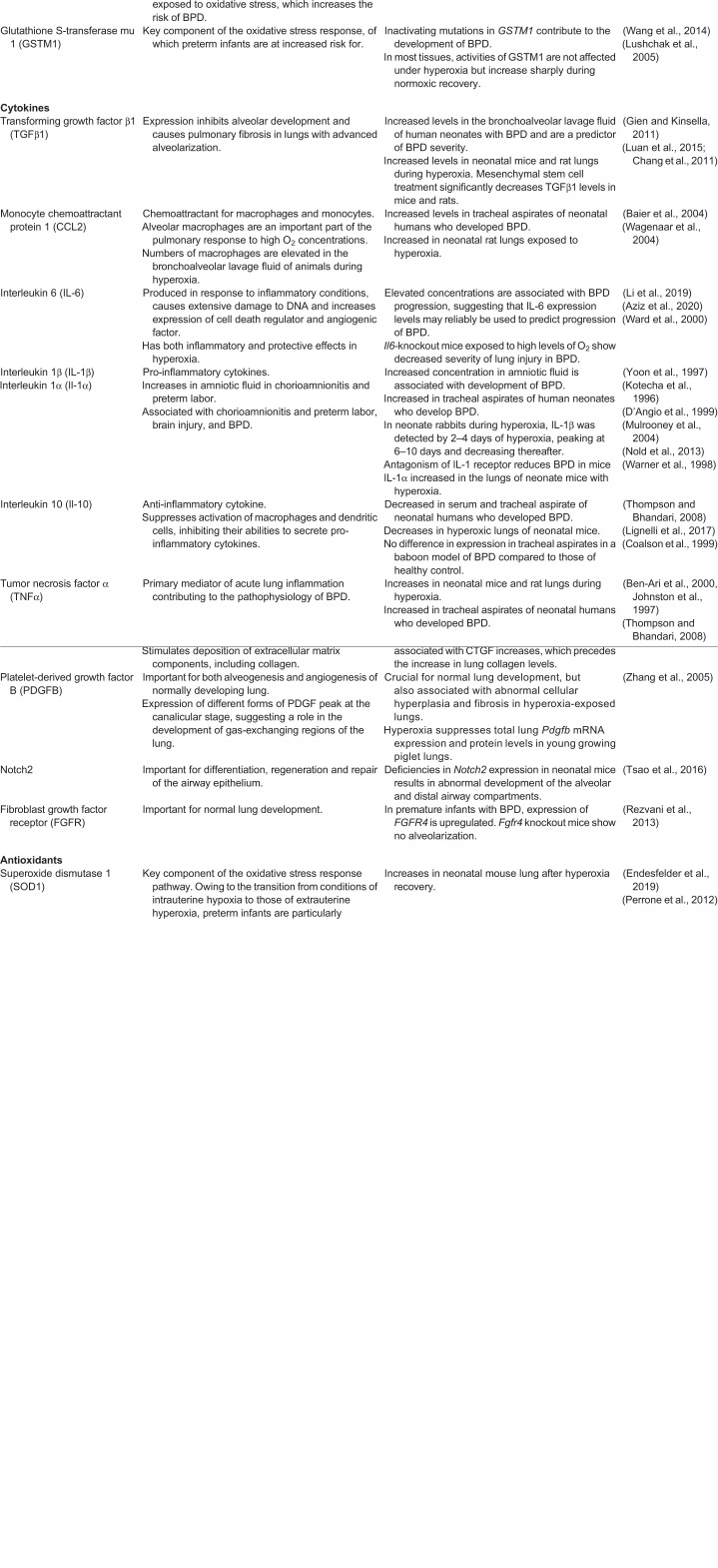
**Proteins of interest in hyperoxia-induced BPD**

Another cytokine affected under hyperoxic conditions, and consistently cited in the literature, is interleukin-6 (IL-6). IL-6 is produced in response to inflammatory conditions, causes extensive damage to DNA, increases cell death regulator and angiogenic factor expression, and has both inflammatory and protective effects in hyperoxia (Ward et al., 2000). IL-6 is elevated in tracheal aspirates of human neonates, who subsequently developed BPD (von Bismarck et al., 2009). Elevated IL-6 concentrations are associated with BPD progression, which suggests that IL-6 expression may be used as a reliable biomarker of BPD (Li et al., 2019). When BPD is modeled in IL-6 knockout mice, the animals demonstrate a decreased severity of lung injury (Aziz et al., 2020). MSC treatment significantly decreases IL-6 secretion in neonatal rats exposed to high levels of O_2_ (Chang et al., 2013).

Several studies of BPD pathogenesis also note the importance of vascular endothelial growth factor (VEGF) in the regulation of pulmonary angiogenesis and alveolar development (Oak and Hilgendorff, 2017; Mariduena et al., 2020; Yang et al., 2015). VEGF is a widely expressed growth factor that is highly expressed in the lung. It is involved in the regulation of vascular growth and development, the stimulation of angiogenesis and the promotion of endothelial survival, all of which are particularly important for the proper functioning of the lungs. Aside from regulating lung vascularization, VEGF appears to be essential for the appropriate development of alveolar tissue (Bhandari, 2010). Hyperoxia triggers a biphasic VEGF response in both human neonates and rodent models, where an initial increase in VEGF may cause lung injury, followed by a drop in VEGF. Subsequent increase of VEGF then promotes angiogenesis and alveolarization, necessary for lung healing and repair (Mura et al., 2004; Bhandari, 2010). Studies have shown that rat pups subcutaneously injected with the VEGF inhibitor VEGF-Trap show impaired alveolarization, especially, in early lung development (Thébaud et al., 2005). Also, rat pups exposed to hyperoxia have significantly lower levels of *Vegf* mRNA in their lungs at 14 days of age (Luan et al., 2015).

Inflammation and changes in angiogenic signaling are not the only consequences of hyperoxia. The release of pro-inflammatory cytokines and dysregulation of angiogenesis leads to significant cellular damage, which is evident in histology and transcription analyses of lung biopsies derived from BPD patients (Dishop, 2010). This damage occurs in the form of apoptosis, an important feature in hyperoxic lung injury, due to highly elevated levels of ROS (Mantell and Lee, 2000). The apoptotic signaling pathway involves a balance between BAX, i.e. the pro-apoptotic member of the BCL2 family, and the anti-apoptotic regulator BCL2, which is crucial to regulate apoptosis. The high levels of ROS upon hyperoxia can overwhelm the antioxidant systems in pulmonary cell types, which then become targets of ROS-induced injury and death. Studies show that hyperoxia-induced lung injury is associated with the death of alveolar epithelial cells, showing features of both apoptosis and necrosis (Das et al., 2004; Tong et al., 2021; Witkowski et al., 2016). Preterm human neonates who have received mechanical ventilation show higher levels of apoptosis, particularly, in epithelial cells (Lukkarinen et al., 2003). A correlation between increasing numbers of apoptotic cells and increasing mRNA levels of *Bax* during hyperoxia was found in neonatal rat lung and, through upregulation of BCL2, administration of MSCs protects against apoptosis in this model (Zhang et al., 2013).

Extracellular matrix dynamics are another important factor in the response of lung tissue to hyperoxia, particularly regarding tissue regeneration and fibrosis. Matrix metalloproteinases (MMPs) are proteolytic enzymes that degrade extracellular matrix components and fibrillar collagen. They have important roles during normal lung development but, in excess, may alter lung remodeling and change lung architecture (Buckley and Warburton, 2002). MMP-9 is implicated in BPD, as studies in preterm human neonates support the argument that hyperoxia leads to increased MMP-9 levels in the lung (Bhandari, 2010; Sweet et al., 2004). Levels of MMP-9 also increase in lungs of neonatal mouse following hyperoxic injury and are associated with impaired alveolar development (Chetty et al., 2008). Compared with lungs from wild-type mice, lungs from MMP-9 knockout mice exposed to elevated O_2_ levels have an increased gas-exchange surface area and are protected from hyperoxia injury (Chetty et al., 2008). During hyperoxia, premature baboons also have increased MMP-9 levels and associated changes in lung architecture, compared with those of premature baboons under normoxic conditions (Tambunting et al., 2005). Upregulated MMP-9 expression during hyperoxia can lead to the activation of secreted glycoproteins, i.e. fibroblast growth factors (FGFs), that have an important role in normal pulmonary lung development and function, as well as in surfactant homeostasis (Rezvani et al., 2013). For instance, FGFR-HFc (HFc, heavy chain hinge and Fc domain of the mouse immunoglobulin) transgenic mice expressing a soluble FGF receptor are more susceptible to hyperoxic conditions following 95% O_2_ exposure compared to normal controls (Hokuto et al., 2004). Moreover, loss of FGFR signaling within type II alveolar epithelial cells leads to larger airspaces, enhanced collagen deposition and increased mortality of mice following lung injury (Dorry et al., 2020). Combined, these findings highlight how hyperoxia triggers a number of responses, which involve cell- and tissue-level signaling pathways that can result in BPD. Model systems, both *in vivo* and *in vitro*, have been crucial for these discoveries and remain instrumental in ongoing research. Additionally, this information is important because the identification of signaling pathways and molecular effects of BPD will aid in the identification of novel biomarkers and the development of therapeutics that reduce the negative effects of this disease.

## Animal models

The majority of hyperoxia-induced BPD studies use mice and rats as their animal model. Both species are born during the saccular stage of lung development, which is characterized by the initiation of surfactant production and terminal airway dilation and vascularization (Berger and Bhandari, 2014). In rodents, the saccular stage begins *in utero* and is completed by postnatal day 5 (PN5) (Berger and Bhandari, 2014). In humans, the saccular stage takes place exclusively *in utero* unless born prematurely. Humans born prematurely, when their lungs are in the saccular stage of development, are at high risk for complications due to pulmonary immaturity and surfactant deficiency (O'Reilly and Thébaud, 2014; Berger and Bhandari, 2014). This highlights a key difference between species. Rodents are born surfactant-sufficient, which limits their risk for respiratory distress during hyperoxic conditions (Berger and Bhandari, 2014). Despite this, rodents are the only animal models born during the saccular stage of lung development and, therefore, are well-suited to mimic the hallmark symptoms of BPD in neonates.

Fundamental differences between rodent species include total lung capacity, alveoli size and differential expression of genes that modulate lung processes (Berger and Bhandari, 2014). There are also strain- and sex-specific differences that need to be accounted for (Leary et al., 2019; Lingappan et al., 2015; Tiono et al., 2019; Dolma et al., 2020). Modeling of hyperoxic conditions in both outbred and inbred rodents, as well as in immunodeficient and immunocompetent ones, can lead to significant variability in results, posing a threat to the clinical applicability of these findings (Leary et al., 2019; Whitehead et al., 2006; Tiono et al., 2019; Will et al., 2019). Results may be irreproducible in outbred strains due to genotypic variations between different animals. In addition, different strains may exhibit distinct levels of gene expression. Whitehead and colleagues analyzed the genetic background of nine different mouse strains and discovered a profound effect on pathophysiologic responses to hyperoxic conditions (Whitehead et al., 2006). However, to study the heterogeneous responses of outbred animals to different drugs or interventions used to treat BPD may be beneficial because they more closely mimic the diverse responses seen in humans. In addition, on a molecular level, the response of rodents to hyperoxia may be protective in certain inbred strains compared to outbred strains, further skewing the translational relevancy of findings. There is also a potential for this to occur in immunocompetent vs deficient rodents, where immunodeficient mice may respond less well to hyperoxia. Therefore, it is imperative to choose a strain showing a response that is most similar to that of a premature infant. For some studies, the use of rats may be beneficial as they are bigger, enabling improved handling during surgery and tissue harvesting, whereas using mice may have the advantage of widespread availability of biological reagents and their amenability to genetic manipulation (O'Reilly and Thébaud, 2014). Therefore, researchers need to consider a number of factors when choosing a rodent model for BPD research.

Unlike rodents, rabbits begin the alveolar stage of lung development *in utero*, similar to a full-term human infant (Richter et al., 2014; Pringle, 1986). Their larger lung capacity makes them better suited than rodents for the study of respiratory diseases affecting premature infants. However, since alveologenesis has already begun in the full-term rabbit, it may not be as well-suited to study this process. Compared to rodents, rabbits are costly and, in the United States, are regulated and protected by the US Department of Agriculture, which means that strict requirements and guidelines are in place to ensure they receive proper handling, care and treatment. However, when researchers need a larger model to study the effects of hyperoxia, rabbits do offer a suitable alternative when the use of larger non-human primates is impossible (D'Angio and Ryan, 2014).

Large animals, such as baboons and lambs are advantageous for studies of hyperoxia-induced lung damage, as they offer the opportunity to study disease progression over an extended time period, and to examine long-term effects of treatment. Pregnant animals can be induced, so that their babies are born prematurely during the saccular stage of lung development and can be placed on a mechanical ventilator at O_2_ levels similar to that delivered to human preterm infants (Albertine, 2015). Because of these attributes, large animals are best-suited for studies of hyperoxia-induced lung injury. Unfortunately, large animals are very expensive, require dedicated infrastructure and, with 150 days for lambs and 168 days for baboons compared to 21 days for rodents, have much longer gestation periods. On the basis of this information, researchers must carefully consider the stage of lung development, ability to manipulate the animal model, its size, cost and gestation time. Smaller animals (mice, rats, rabbits) are advantageous because of their short life cycle, low cost and the ability to manipulate their genomic and molecular pathways (Albertine, 2015; Ambalavanan and Morty, 2016). Large animal models (lambs, baboons) are advantageous owing to their ability to mimic human preterm lung disease because of similarities in lung size and the capability of replicating chronic respiratory conditions over extended periods of time (Albertine, 2015). The establishment of reliable and consistent standardized methodologies in small animal models of BPD, followed by replication in studies using large animals, should inform clinicians of the most-effective therapies for BPD in neonates. Despite their many advantages, certain questions cannot be fully addressed by research on animal models alone and require human-derived model systems.

## *In vitro* models

To gain a deeper understanding of the molecular underpinnings of BPD pathogenesis, *in vitro* models of hyperoxia were developed that incorporate cells derived from human and rodent fetal lung tissues. Table 2 shows an overview of the different types of models as well as sources from which cells and tissue are commonly derived. These include 2D cultured cells or 3D-organotypic lung co-cultures as well as *ex vivo* anatomical scaffolds or models derived from live tissue or 3D-printed using biomimetic materials (Sucre et al., 2020; Montigaud et al., 2019; Möbius et al., 2018; Jiang et al., 2018; Leeman et al., 2019; Montemurro et al., 2006; Sucre et al., 2018; Knoll et al., 2013). Fetal primary epithelial cells isolated from human or rodent lung tissues can be cultured together with lung-derived fibroblasts or MSCs to study the effect of hyperoxia regarding the expression of transcription factors associated with pulmonary development. Sucre et al. have described a possible mechanistic link between hyperoxia and increased expression of Wnt5A – a protein involved in lung development – when human primary alveolar type II cells and fibroblasts were co-cultured under hyperoxic conditions (Sucre et al., 2020). Through this and other studies, it is now established that the Wnt5a signaling pathway is a mediator of chronic lung disease (Newman et al., 2016; Li et al., 2020; Lingappan and Savani, 2020). Because on molecular and transcriptional levels the above models show a response that is similar to that in animal models, they serve as a complementary resource to evaluate and manipulate the signaling pathways involved in the perturbation of lung development (Torday and Rehan, 2007; Asikainen et al., 2005a; Blackwell et al., 2011). Response to certain inhibitors and treatments can first be tested in these *in vitro* models, followed by implementation in animal models. For example, Zhang and colleagues have demonstrated that isolated hyperoxic rat alveolar cells treated with the autophagy inducers rapamycin or LiCl show restored autophagy flux and increased survival of cells (Zhang et al., 2020). These types of study help to reduce the number of animals initially required to obtain the desired therapeutic response. *In vitro* model systems are also beneficial because they allow for the evaluation of parameters associated with epithelial-to-mesenchymal transition (Greer et al., 2014), cell migration (You et al., 2019) and angiogenesis (Zhang et al., 2018). The influence of sex on BPD severity has also been investigated by using a 2D *in vitro* model of hyperoxia (Zhang et al., 2017). Zhang and colleagues exposed human umbilical vein endothelial cells (HUVECs) derived from males and females to hyperoxic (95% O_2_) and normoxic conditions (21% O_2_) for up to 72 h. They discovered that mRNA and protein levels of the secreted cytokine growth and differentiation factor 15 (GDF15) – that plays a role in cell proliferation, angiogenesis and apoptosis – is upregulated in male-derived compared to female cells. Zhang et al. were able to reproduce these findings *in vivo* by exposing male and female C57BL/6 mice to the same conditions.

**Table 2.**
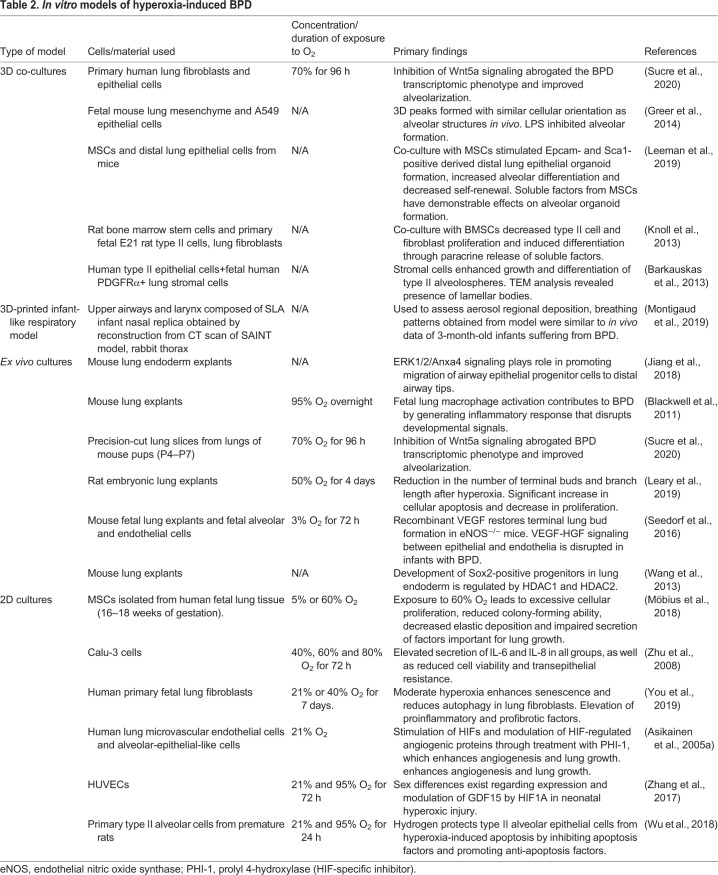
***In vitro* models of hyperoxia-induced BPD**

Another benefit to the use of *in vitro* models for the study of BPD pathogenesis is that the stages of embryogenesis seen within the developing lung can be mimicked in culture. This allows for the identification of transcription factors affected by hyperoxia at each stage of development, which can further inform clinicians on the best course of action for treatment (Barkauskas et al., 2017). To mimic embryogenesis, bioengineered stem cells, such as induced pluripotent stem cells (iPSCs) are utilized as they can undergo a step-wise differentiation, using growth factors to promote pulmonary development. Our laboratory as well as others have successfully differentiated iPSCs to distal lung phenotypes that mimic type I and type II alveolar cells (Mitchell et al., 2017; Wang et al., 2020; Alysandratos et al., 2020 preprint). Type I and type II alveolar cells are implicated in the functional regeneration of the distal lung after damage and are an excellent source for modeling hyperoxia-induced BPD (Jacob et al., 2017; Heo et al., 2019). In addition to studying cell development using cell culture, explant lung culture is another option to investigate specific pathways and novel interactions affected by hyperoxic conditions. Lung tissue explants can be studied before, after or during hyperoxia (Karekla et al., 2017; Asikainen et al., 2005b), and are used to study branching morphogenesis (Esquibies et al., 2008; Esquibies et al., 2014; Seedorf et al., 2016). Lung tissue explants can also be used to study the effects of transcription factors on lung repair pathways. In one study, researchers exposed fetal baboon explants to high (95%) levels of O_2_, followed by treatment with prolyl hydroxylase domain-containing protein inhibitors with the aim to enhance angiogenic responses within the injured lung (Asikainen et al., 2005b). Their findings demonstrate increased expression of hypoxia-inducible factors 1α and 2α (HIF1A and HIF1B, respectively), as well as VEGF and platelet-endothelial cell adhesion molecule 1 (PECAM1), and are important because they provide a potential therapeutic option for the enhancement of lung growth during BPD. Overall, *in vitro* models are an attractive alternative to the use of *in vivo* models as they allow easy manipulation and direct visualization of lung development by using light or fluorescent microscopy (Wang et al., 2013).

In summary, *in vitro* models meet the need to reduce the use of animals in research, and provide an easily accessible platform in which to dissect the currently less understood molecular consequences of hyperoxia and to screen candidate therapeutics for treatment of BPD (Wu et al., 2018; Leeman et al., 2019; Knoll et al., 2013). Knowledge gained from these customizable models will allow investigators to manipulate developmental pathways within the lung, in order to improve clinical outcomes associated with BPD. However, these models should serve as a complement to *in vivo* studies, rather than a substitute, as they do not fully recapitulate the complexities of the *in vivo* microenvironment. Additionally, as evidenced from the studies cited in Table 2, considerable variability exists between different types of model, the source of tissue/cells and the duration and concentration of O_2_ exposure used for hyperoxia experiments, i.e. caution should be taken when comparing these findings to *in vivo* conditions. Therefore, the continued use of animal models of hyperoxia-induced BPD remains essential for successful clinical progress.

## Differences in methodologies to model BPD in animals

To induce hyperoxic lung damage, animals are exposed to high levels of O_2_ (60–95%) for a set period of time. As discussed previously, studies of animal models vary regarding species, O_2_ concentration and duration of exposure, and lung injury measures (see Table 4). In rodents and rabbits, hyperoxia is achieved in a regulated chamber that mixes air and O_2_. Some studies also employ mechanical ventilation to induce lung damage in small and large animals, with the aim to mimic the conditions experienced by at-risk neonates in the NICU. By itself, mechanical ventilation can greatly affect the inflammatory response within the lung (Helmerhorst et al., 2017). Therefore, the two methods for O_2_ delivery described above are not interchangeable. Treating them as such would pose a threat to the standardization of methodologies, which is essential for a translationally relevant evaluation of hyperoxia-induced lung damage. This should be carefully considered when using a protocol that adopts a combination of the two types of O_2_ exposure.

**Fig. 3.**
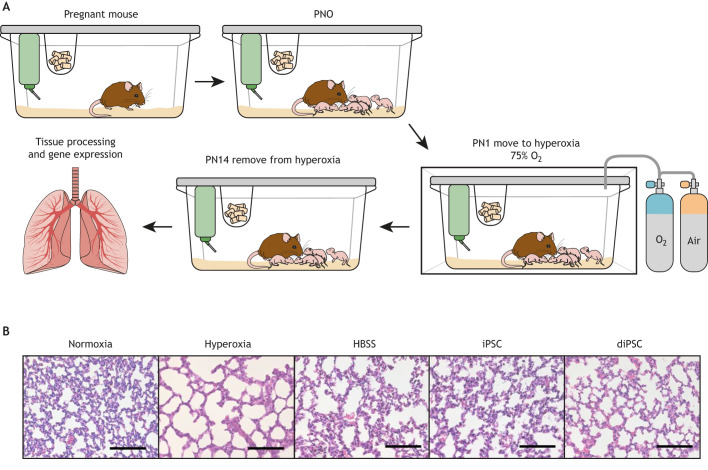
**Development and analysis of a hyperoxia-induced BPD mouse model.** Developed from Mitchell et al., 2020 (A) Mice born under normoxic conditions. At postnatal day 1 (PN1), the cage is transferred to a hyperoxia chamber with a humidified atmosphere and O_2_ regulated to 75%. The dam is fed and given water *ad libitum*, and the wet nurse is changed every 48 h to ensure welfare by limiting the noxious effects of hyperoxia on adult animals. At PN14, the cage is removed from the hyperoxia chamber, and lung tissue from the pups is harvested and processed to analyze changes in morphology and gene expression. (B) Histology images of lungs obtained from neonatal mice under normoxic and hyperoxic conditions, and of lungs from neonatal mice under hyperoxic conditions treated with induced pluripotent stem cells (iPSCs) or iPSCs differentiated to an alveolar-like phenotype (diPSCs). The images show that lungs from animals under hyperoxic conditions exhibit alveolar simplification and that iPSC treatment restored lung morphology to that of lungs under normoxic conditions. diPSC treatment did not completely reverse alveolar simplification but showed improvement compared to non-treated lungs from animals under hyperoxic conditions. Hanks balanced salt solution (HBSS) was used as vehicle control in the treatment experiments. Scale bars: 100 μm.

Silva et al. found that studies published between 2012 and 2015 described 41 different O_2_ exposure protocols to induce lung damage in animal models (Silva et al., 2015). Silva's review and Table 3 highlight that even these small subsets of studies substantially vary in the degree and duration of hyperoxia, and in the definition of lung injury associated with BPD (Silva et al., 2015), which greatly affects the translatability of findings. The expression of genes affected during hyperoxic conditions will vary significantly if the duration and concentration of O_2_ exposure varies. For results to be reproducible, studies should focus on finding the optimal O_2_ concentration and exposure time to mimic BPD symptoms without causing lethal injury. Based on our previously published findings, we have shown that exposing mice at PN1 to 75% O_2_ for 14 days is sufficient to induce alveolar simplification and septal thickening similar to those seen in a premature infant suffering from BPD (Fig. 3), corroborating findings by others (Mitchell et al., 2020; Popova et al., 2014; Kindermann et al., 2019; Schmiedl et al., 2017). Nardiello et al. exposed C57/BL6 mouse pups to 85% O_2_ for 14 days and used design-based stereology to analyze lung tissue samples (Nardiello et al., 2017b). They showed that – compared with pups under normoxic conditions – this level of hyperoxia sufficiently recapitulates the features of BPD, as it causes significant alveolar simplification, i.e. a 69% decrease in alveoli number and alveolar density, a 37% decrease in gas exchange area, and 25% thickening of the septal wall. Although exposure of mouse pups to 85% O_2_ successfully induced a BPD phenotype, this dose might not be ideal when evaluating the effectiveness of therapeutic interventions. Nardiello et al., therefore recommend to evaluate the intervention by reducing the O_2_ concentration to 60%, if said intervention is unable to reverse the damage caused by 85% O_2_ (Nardiello et al., 2017b). This highlights the idea that severe disease phenotypes, like the murine BPD caused by exposure to 85% O_2_, might not be the most appropriate vehicle to test the effectiveness of therapies, and that researchers must carefully select and evaluate their experimental parameters.

**Table 3.**
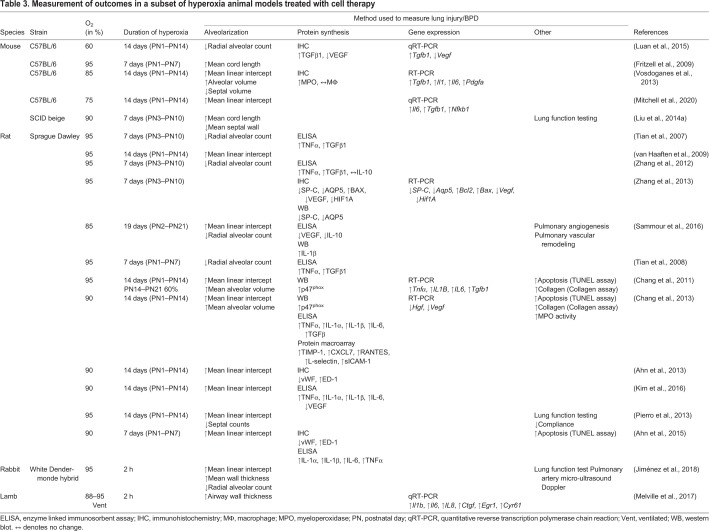
**Measurement of outcomes in a subset** **of hyperoxia animal models treated with cell therapy**

**Table 4.**
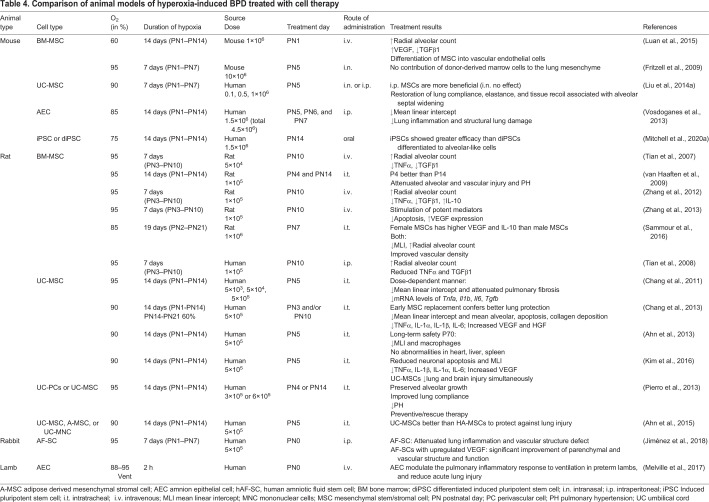
**Comparison of** **animal models of hyperoxia-induced BPD treated with cell therapy**

In addition to the lack of agreement regarding the optimal O_2_ concentration to elicit appropriate lung damage, most current studies did not assess lung function after damage. BPD is no longer defined on the basis of morphological changes alone, but on the need for supplemental O_2_ to live (Ibrahim and Bhandari, 2018). Accurate functional assessments in rodents are difficult as they are small and anatomically different compared to human neonates. However, accurate and clinically valid morphological and functional assessments are necessary for translationally relevant comparisons between species (Hoymann, 2012). Examples of tests used for functional assessments of the lung include whole-body plethysmography and forced oscillation. The whole-body plethysmography method involves an inner restraint chamber, in which the rodent is placed and positioned within a nose cone attached to a pneumotachograph, a flow-resistive device that quantitatively measures airflow. It contains plethysmography chambers with ports for bias flow, calibration and leak testing (Lofgren et al., 2006). It also allows to monitor changes in airway pressure and temperature at the nose of the rodent. Forced oscillation techniques are employed by using a flexiVent system made for small rodents. This method involves sedation and intubation of animals, followed by testing of lung function upon the application of forced oscillation signals over a set period. An example of this method was performed in neonatal rat pups exposed to three different concentrations of O_2_ (fractions of inspired O_2_: 0.6, 0.8, and 1.0) over a total of 18 days. Results showed altered tissue stiffness and reduced lung compliance following injury, as well as alveolar simplification, increased ACTA2 content in pulmonary vessels and fewer pulmonary arterioles (Greco et al., 2019). This study demonstrates that functional lung assessments are associated with structural changes within the lung and are, thus, a necessary parameter to consider when comparing results from animal models to premature infants.

## Lung injury measures in animal models

A BPD diagnosis is based on the need for supplemental O_2_ for at least 28 days after birth, whereas its severity is graded according to the respiratory support the infant requires at 36 postmenstrual weeks of age (Nardiello et al., 2017a). The clinical definition of BPD, thus, does not consider the structural damage hyperoxia causes to a developing lung. The goal of many BPD animal model studies is to explore interventions, with the aim of promoting lung growth and alveolarization. Hyperoxia-induced BPD models should show a combination of changes in lung morphology, and changes in the expression of relevant genes that mimic the multifactorial etiology of BPD. Currently, there is no standardization of lung injury measurement when exploring new interventions for BPD in animal models. Therefore, it is important to effectively quantify changes to the lung structure, especially the size and number of alveoli, the gas-exchange surface area, the vascularization of the lung, and the thickness of the septal walls (Nardiello et al., 2017a). Such quantitative information about lung architecture is necessary to assess pathological alterations as well as treatment effects, and to allow statistical comparisons among experimental groups (Ochs and Mühlfeld, 2013). A decrease in the available surface or an increase in the thickness of a barrier can result in functional impairment, leading to problems, such as emphysema and fibrosis (Mühlfeld et al., 2013). Additionally, transcriptomic and cellular alterations caused by BPD indicate disease severity and the standardization of markers to evaluate these changes in animal models is necessary to make the appropriate comparisons to neonates. Several of the current studies focus solely on morphological changes associated with hyperoxia-induced lung injury and neglect to highlight the molecular implications of BPD.

### Morphological assessments of the lung following hyperoxic damage

In most studies, the mean linear intercept (MLI) or radial alveolar count (RAC) are techniques used for direct measurements of the distance between adjacent walls of an alveolus by means of a slide rule. MLI is used as an indirect method to measure air space enlargement, which includes both alveoli and alveolar ducts. RAC is a measure of the number of alveoli or degree of alveolarization within the lung. Unfortunately, there are downsides to using MLI and RAC in BPD animal models, such as inherent bias, especially if investigators are not double-blinded during their assessments. Moreover, dimensions within the distal air spaces are vastly different under physiological conditions compared to when the lungs are fixed for analysis (Knudsen et al., 2010). Distortion of the lung structure can occur during tissue processing, potentially introducing artifacts associated with non-uniform tissue dehydration and rehydration (Schneider and Ochs, 2014; Silva et al., 2015). Accurate morphological assessments in rodents is particularly difficult because of their small size and inability to mimic chronic respiratory conditions. Thus, the degree of lung inflation as well as length of tissue fixation need to be standardized to eliminate discrepancies in MLI and RAC findings.

Quantitative assessment of lung structure is essential to thoroughly understand the changes that occur in a diseased state (Mühlfeld et al., 2013). Microscopic study of the internal structure of the lung is an indirect measurement of morphometry, whereby a small section of the lung is analyzed; this also can potentially introduce bias. Recommendations have shifted to the use of design-based stereology to assess changes to the lung morphometry. The American Thoracic Society and the European Respiratory Society have both defined the standards for performing quantitative assessments of lung structures by using this method (Hsia et al., 2010), which involves measuring 2D sections of tissue to characterize physical properties of irregular 3D objects. (Weibel et al., 2007). The use of computed tomography and magnetic resonance imaging may also help to identify and distinguish pulmonary abnormalities following hyperoxia-induced lung damage (Westcott et al., 2019). A combination of direct measurements such as MLI and RAC, together with indirect measurements like design-based stereology, will greatly enhance the reliability and reproducibility of morphological findings, as well as eliminate inherent bias and, thus, improve the translational potential of hyperoxia-induced animal models of BPD.

### Molecular assessments of the lung following hyperoxic damage

Both morphologic and molecular assessments are necessary to ascertain the full effects of hyperoxia in animal models of BPD. Table 1 highlights the genes differentially expressed in neonatal humans and neonatal animal models, showing that BDP is multifactorial in nature. Therefore, it is imperative to develop an animal model that exhibits the same degree of lung injury as a preterm infant on the transcriptomic and molecular level. The majority of pre-clinical studies of hyperoxia focus on morphological parameters to assess lung damage, which is not always representative of the changes that occur within the lung. Analysis of gene expression in the lung during hyperoxia will provide valuable information as to when molecular damage occurs – because this often precedes physical damage. This will provide a source of comparison to genes affected in the neonate, increasing the potential translational significance of the findings to better inform researchers and clinicians as to when therapeutic interventions will be most effective.

## Stem cells as a potential treatment for BPD

Although several different therapeutics have been tested for the treatment of BPD (Principi et al., 2018), the most promising – based on results from pre-clinical studies – are exogenous stem cells, which can be delivered via several different routes intravenously, intraperitoneally, intramuscularly, intraorally or intranasally. Stem cells are an attractive therapeutic option owing to their excellent anti-inflammatory properties and regenerative abilities mediated by their paracrine effects on neighboring cells (Namba, 2019). Progenitor-like stem cells are depleted in infants suffering from BPD, making exogenous administration of allogenic or autologous stem cells beneficial (Borghesi et al., 2013). The types of stem cell utilized include amnion epithelial cells (AECs), MSCs and induced pluripotent stem cells (iPSCs) (Möbius and Thébaud, 2015). Pre-clinical studies have shown that delivery of stem cells can protect the lungs and restore damage in different animal models of BPD (Table 3).

### Amnion epithelial cells

Amnion epithelial cells (AECs) are pluripotent and derived from the placenta. In both mouse intraperitoneal and lamb intravenous AEC administration models, the AECs attenuated damage caused hyperoxia-induced inflammation, and improved lung function and structure (Melville et al., 2017; Vosdoganes et al., 2013). Clinical trials are currently in progress to assess their efficacy in the treatment of BPD in neonates (Baker et al., 2019; Lim et al., 2018).

### Mesenchymal stromal/stem cells

MSCs are multipotent stem cells that can be isolated from many tissue types (Ee and Thébaud, 2018); MSC therapy is the most extensively studied cell therapy for BPD in hyperoxia models. This cell therapy focus has been placed on MSCs owing to their pleiotropic effects of immune modulation, angiogenesis and anti-oxidant activity – key hallmarks of BPD (Ee and Thébaud, 2018; Davis et al., 2012; Kuchroo et al., 2015; Dey et al., 2012). A concern with the use of certain stem cells is their ability to form non-cancerous tumors called teratomas upon *in vivo* administration (Hentze et al., 2009; Gutierrez-Aranda et al., 2010). However, MSCs are safe and non-teratogenic, making them an attractive therapeutic option.

Results from studies in which MSCs were administered to animal models of hyperoxia-induced lung injury have been promising. MSCs administered either intravenously, intraperitoneally or intratracheally have mitigated neonatal lung injury – as shown by decreased lung inflammation (O'Reilly and Thébaud, 2014), prevention of vascular damage and of alveolar growth arrest (Aslam et al., 2009), inhibition of lung fibrosis (Tropea et al., 2012), and improvement of exercise tolerance (Hansmann et al., 2012; Zhang et al., 2012).

### Induced pluripotent stem cells

Another type of stem cell investigated as a potential therapeutic are induced pluripotent stem cells (iPSCs). These cells can differentiate into all three embryonic germ layers and have immunogenic properties similar to those of MSCs, but show more potent immunomodulatory effects *in vitro* (Schnabel et al., 2014). iPSCs are capable of unlimited *in vitro* proliferation and can be differentiated into virtually any cell type of the body.

iPSCs have been found to attenuate acute lung injury caused by mechanical ventilation, by suppressing oxidative stress, acute inflammation and apoptosis (Liu et al., 2014b). Data from our laboratory has shown that iPSCs, when administered intraorally after exposing mice to hyperoxic conditions (75% O_2_ for 14 days), attenuate alveolar simplification (Fig. 2A) and restore lung morphology to that seen in normoxic control animals (Fig. 2B) (Mitchell et al., 2020).

A limitation of transplanting undifferentiated iPSCs is their potential to form teratomas *in vivo*, whereas differentiated iPSCs do not, as previously published by our laboratory (Mitchell et al., 2019). Studies from our group and others have evaluated the effect of transplanting iPSCs that had differentiated – i.e. diPSCs – into an alveolar-like phenotype in attenuating hyperoxia-induced lung injury (Mitchell et al., 2019, 2017; Shafa et al., 2018; Huang et al., 2014). Our lab showed that, although alveolar-like diPSCs can prevent hyperoxia-induced impairment of lung function and alveolar growth in mice, they are not as efficacious as undifferentiated iPSCs (Fig. 2B) (Mitchell et al., 2020; Shafa et al., 2018).

Stem cell-based therapy for the treatment of hyperoxia-induced lung disease is an exciting and evolving field. Studies have shown that the anti-inflammatory effect of stem cells benefit lung repair; however, there are still inherent risks associated with their use (Chang et al., 2014; Mitchell et al., 2020; Shafa et al., 2018; Liu et al., 2014b). Certain cell types can transform into inappropriate phenotypes that may lead to cancer, and may have pro-arrhythmic side effects (Toh et al., 2018). Unfortunately, as with animal models, their variability in terms of cell type, source species and route of administration as well as the age of the recipient at treatment affect outcomes. MSCs isolated from bone marrow, umbilical cord, Wharton's jelly and adipose tissue have been tested in hyperoxia-induced BPD animal studies together with AECs, iPSCs and differentiated iPSCs. These studies show great promise and some groups are testing MSCs in clinical trials for safety and possible efficacy (Ahn et al., 2017; Powell and Silvestri, 2019). However, it is imperative that the field standardize these studies to determine which combination of parameters best mimics the BPD seen in human neonates, and to test possible treatment options.

## Conclusions

Considerable differences in animal models and methodologies used to analyze the effects of hyperoxia-induced lung damage exist, hampering the potential to develop reliable and effective therapies for the treatment of BPD in neonates. *In vitro* models have the potential to address many of the shortcomings associated with current animal models, by revealing the cellular and molecular pathways involved in the progression of BPD. 3D-organotypic models are able to replicate embryogenesis of the lung, and provide excellent platforms for the safe and efficient testing of different therapeutics.

Based on a thorough analysis of the current hyperoxia literature, rodent models of BPD seem best-suited for the initial study of hyperoxia-induced lung damage and potential therapeutic treatments (Box 1). Not only are they suitable in terms of cost and gestation length, but they more closely mimic the stage of lung development seen in a preterm infant. Based on our previously published study, standardized models should be developed – implementing at least 75–85% O_2_ for 14 days – to achieve the morphological alterations associated with BPD, such as alveolar simplification, fibrosis and septal wall thickening (Mitchell et al., 2020). Even though stem cell therapy shows promise in treatment of BPD, there is still a long way to go in terms of safety and efficacy in clinical trials. However, MSCs and iPSCs are consistently cited in the literature as having beneficial effects. The route of administration for cell-based therapies should be one that successfully delivers cells directly to the lung for the greatest tissue regeneration benefits. Our own work showed that the intratracheal or intraoral routes are most effective in the amelioration of lung damage within animal models (Mitchell et al., 2020). Analysis of changes to gene expression during hyperoxia-induced lung damage in rodent models should be comprehensive and mirror the genes affected in the neonate to make direct comparisons. Techniques standardized and validated in rodent models should be implemented in large animals, such as sheep, to evaluate reproducibility and potential for clinical translation. Standardized functional assessments of lungs following damage should also be included in preclinical studies, as BPD is no longer solely defined on the basis of morphological changes. *In vitro* systems can complement animal models to study the molecular mechanisms and pathways related to BPD progression, and for development and validation of potential therapeutics. Once a rigorous set of guidelines and methodologies are established and efficiently reproduced, the findings in both pre-clinical animal and *in vitro* models can be translated into potential therapeutic options for neonates suffering from BPD.

**Box 1. Recommendations for the standardization of current models of hyperoxia-induced tissue damage**

• Develop a set of guidelines to improve animal reporting that will encourage the development of rigorous experimental design standards, similar to the ARRIVE essential 10 checklist, a minimum set of guidelines that need to be reported to improve research involving animals. This will maximize the reproducibility, quality and reliability of published research (see Table 5).

• Determine appropriate model species based on translational relevancy and cost (small rodent vs large animal).

• Establish the appropriate O_2_ concentration and duration of exposure to elicit morphological and genotypic changes in *in vitro* and *in vivo* models of bronchopulmonary dysplasia.

• Establish appropriate methods of O_2_ administration, e.g. mechanical ventilation vs regulated chamber, or both.

• Standardize techniques for morphological analysis of the damaged lung, e.g. mean linear intercept vs radial alveolar count vs design-based stereology.

• Include functional assessments of the lung post damage.

• Analyze changes in gene expression by investigating markers affected in neonates suffering from bronchopulmonary dysplasia.

• Standardize the source of cells for *in vitro* models and therapeutic studies, e.g. stem cells vs primary cells from the lung.

• Standardize the route and frequency of stem cell administration in *in vivo* treatments.

**Table 5.**
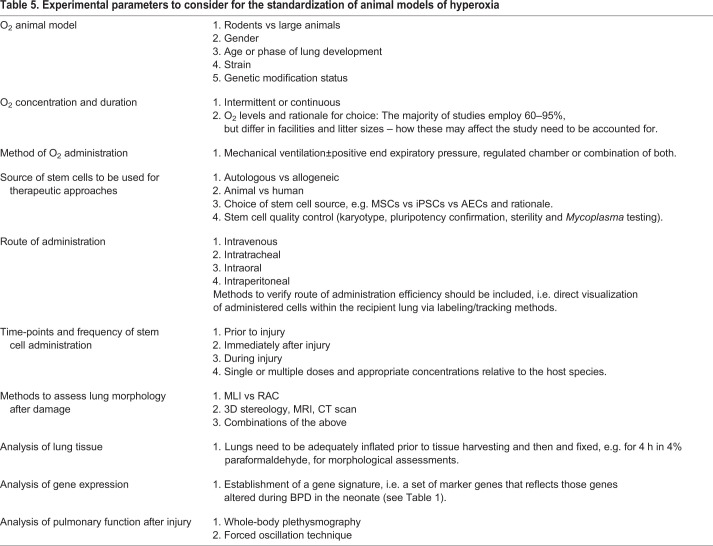
**Experimental parameters to consider for the standardization of animal models of hyperoxia**

In conclusion, considerable work still needs to be accomplished to improve the current models of BPD. However, substantial progress has been made in the identification of molecular and cellular pathways, as well as transcription factors and cytokines implicated in BPD pathogenesis in both *in vivo* and *in vitro* models. This information has contributed to a deeper understanding of disease progression and lung development overall. Implementation of a multidisciplinary approach, involving researchers, developmental biologists, clinicians and pathologists, will help to advance the discovery of viable therapeutic options and appropriate interventions for BPD and, potentially, other chronic respiratory diseases that currently lack sufficient treatment options.
